# Scientific education early in the curriculum using a constructivist approach on learning

**DOI:** 10.1007/s40037-013-0072-1

**Published:** 2013-08-23

**Authors:** M. W. C. Vereijken, M. Kruidering-Hall, P. G. M. de Jong, A. J. de Beaufort, F. W. Dekker

**Affiliations:** 1 Center for Innovation in Medical Education, Leiden University Medical Center, PO Box 9600, 2300 RC Leiden, the Netherlands; 2 Department of Cellular and Molecular Pharmacology, University of California, San Francisco, CA USA; 3 Department of Clinical Epidemiology, Leiden University Medical Center, Leiden, the Netherlands

**Keywords:** Undergraduate medical education, Scientific education, Research and education

## Abstract

Physicians need to stay up-to-date with new developments in their field of expertise. This expectation has been made explicit by competency-based educational outcomes in the domain of scholar in the Dutch blueprint. There is a great diversity in teaching methods that aim to achieve a better understanding of scientific knowledge. Applying a constructivist approach to learning in acquiring research competencies we wonder how a research-intensive course is evaluated early in the curriculum and what learning gain students perceive. In a collaborative research-intensive course, the class of 300s-year students rated the quality of 150 preselected randomized controlled trials (RCT) using JAMA Users’ Guides, and the pharmaceutical advertisements in which they were referenced. Each student rated two RCTs. Data were analyzed to answer a relevant research question. After the course students completed an evaluation survey. We did this in five consecutive years to capture student experience in relation to fostering a scientific mindset (*n* = 1,500). In addition we studied outcome of this scientific mindset as scientific output (publications) in journals. Survey data indicate that it is feasible to successfully implement a research-intensive course based on a large cohort using a constructivist paradigm early in the curriculum. Students consider it challenging and report high learning gain in several domains. Aggregated data have even led to four publications in journals. Implementing an active learning research experience early in the curriculum can foster student attitudes, provided the level of difficulty correctly matches the learners’ prior knowledge. Further research is required to determine how to improve these active research curricula to maximize impact on learners.

## Introduction

Physicians need to stay up-to-date with advancements and new developments in their field of expertise. This expectation has been made explicit by competency-based educational outcomes in the domain of scholar in the blueprint from the Dutch Federation of University Medical Centres (NFU) [1]. The NFU’s expectation suggests that scientific education is an essential part of the medical curriculum. In other parts of the world research competencies for medical students have been explored and specified as well [2, 3]. To allow future physicians to better appraise research findings and contribute to furthering the clinical field by conducting research, medical students need to understand how to conduct research [4].

There is a great diversity in teaching methods that aim to achieve a better understanding of scientific knowledge, increased scientific skills and changed attitudes towards science in students [5, 6]. Medical schools integrate research and education in different ways. Active, authentic learning is shown to be an effective way to acquire a higher level of learning skills [4]. It is rooted in the constructivist approach to learning, referring to the principle that the learner builds his/her knowledge depending on his/her prior knowledge, in an active, goal-oriented, meaningful manner and often together with peers [7]. An example of constructivist learning in clinical undergraduate courses is small-group teaching in which students first come up with what they already know about a subject, apply that in another context (e.g. patient problem) and then find out what knowledge they have to acquire to reach their learning goal (e.g. come up with a good diagnosis). Developing courses that acknowledge the principles described above in the role of scholar [1] seems challenging [8].

There is a high contrast to building knowledge in a constructivist approach as described above and everyday practice in contemporary research courses in medical curricula. In traditional curricula students learn about research early in their studies by hearing about it passively in a lecture course or at best in a teacher-centred exercise or practicum. These settings offer a limited authentic context and students do not conduct real research themselves until much later in the curriculum. In addition to the drawback of the student not having an authentic role, this teacher-centred model of science education early on in the curriculum is labour intensive and takes the researcher away from his/her research and adds to the perceived tension between the research mission and educational mission of the university [9]. To facilitate meaningful, authentic learning, it is important for students to conduct real research.

To implement a constructivist approach, one needs to actively involve students in authentic research-intensive courses. However, such research-intensive courses are usually only offered at the end of the curriculum. We postulate that doing research much earlier would foster a scientific mindset, referring to a structured way of critical thinking, right at the start of medical education. In addition, it could have a highly motivational effect on students up to graduation. Therefore, we designed a research-intensive course for second-year medical students, based on constructivist theory and addressed the following questions:

1. Can a research-intensive course take place early in the curriculum using a constructivist approach to learning with a large cohort of students?
2. Does actively conducting research in a course early in the curriculum foster a scientific mindset in the learner, as perceived by students?
3. What is the level of authenticity in such early research, measured by publication of the results in journals?

## Methods

We designed and implemented such a research-intensive course for second-year medical students. With minor modification we gave the course in five subsequent years to a total of 1,500 students and evaluated the outcomes.

### Course description

In the second year of our medical curriculum students participate in a 3-week course in scientific training (evidence-based medicine). The course focuses on several specific scientific skills and aims to foster a scientific mindset and encourage participation in science. It offers training in different study designs, the basics of statistical analysis, as well as presentation skills through presenting the results of a published paper to colleagues in small-group meetings.

Each year we selected 150 randomized controlled trials (RCTs) mentioned in drug advertisements and provided students with the formulated research question: ‘What percentage of these trials accurately support the claims made in the advertisement?’ Introductory lectures were on RCTs as research design, philosophy of science, bias, confounding and regression, and critical reading. Students practised critical reading and discussed a single RCT in a small-group setting. Key issues such as randomization, treatment allocation, and blinding were addressed. After this meeting, each student received two out of the 150 original RCTs from peer-reviewed journals, to rate according to the JAMA Users’ Guides on how to read a paper on effect of therapy [10]. Thus, with four students per RCT, a class of 300 students rated 150 RCTs and judged their reliability and correctness in relation to the advertisement for which they were used. The students entered the data in an online database. Statistical analyses were performed by students and the results discussed in class. The student-researchers concluded that a solid RCT does not automatically support the claim made in the advertisement, and thus that critical appraisal is a necessary skill for medical doctors. Details on analysis and students’ conclusions have been published [11].

After the 3-week course, the students completed a student evaluation survey on paper to measure several constructivist aspects (e.g. I could relate the assignments to (patient) problems in my future daily practice) of the course and perceived learning gain (I have improved my skills to read critically) in scientific skills and mindset. We collected the surveys from 2008 until 2012. The items are measured on a five-point Likert scale from ‘completely disagree (1)’ to ‘completely agree (5)’. To judge the quality of the scientific output we counted publications on aggregated data in journals.

## Results

The question on what percentage of trials accurately support the claims made in the advertisement is answered by three cohorts of students and published in journals in medicine and medical education [12–15]. In all four publications participating medical students were first author. The authors wrote the articles after the course as an extracurricular activity.

In 5 years, 1500s-year medical students took this course. In total, 1,266 completed a questionnaire at the end of the course (84.4 % response rate). The results on several constructivist aspects of the course and perceived learning gain in scientific skills and mindset are shown in Table 1. Students reported that they improved their ability to read critically (mean score 3.8) (on a Likert scale from 1 to 5), and also that the course did contribute to fostering a scientific mindset (mean score 3.6).

**Table 1:** Results on the self-evaluation questionnaire (Likert scale scores 1–5) on constructivist aspects of learning and learning gain in scientific skills and mindset (mean (SD))

	2008 *n* ^*^ = 240	2009 *n* ^*^ = 243	2010 *n* ^*^ = 277	2011 *n* ^*^ = 252	2012 *n* ^*^ = 254	Total *n* = 1,266
I had enough prior knowledge to do this course	3.6 (0.8)	3.5 (1)	3.8 (0.8)	3.7 (0.9)	3.9 (0.9)	3.7 (0.98)
In the beginning of this course I knew what I had to know and do by the end of the course	2.8 (0.9)	2.7 (1)	3.1 (0.9)	3.0 (1)	3.2 (1)	2.7 (1.16)
I could relate the assignments to (patient) problems in my future daily practice	2.5 (1)	2.5 (1)	2.6 (1)	2.6 (1)	2.9 (1)	2.6 (1.25)
The assignments were challenging	2.4 (0.9)	2.4 (0.9)	2.5 (0.9)	2.7 (0.9)	3.5 (0.9)	2.7 (1.01)
I learned a lot during this course	3.1 (0.8)	3.1 (0.9)	3.1 (0.8)	3.2 (0.7)	3.1 (0.9)	2.9 (0.85)
I have improved my ability to read critically	3.8 (0.8)	3.8 (0.9)	3.8 (0.8)	No data	No data	3.8 (1.05)
Examining RCTs using guidelines was very informative	No data	No data	No data	3.3 (1.1)	3.4 (1)	3.3 (2.21)
I understand the importance of a scientific mind in medical education	3.9 (0.8)	3.6 (1.1)	3.5 (0.9)	No data	No data	3.7 (1.33)
This course contributed to fostering a scientific mind	3.7 (0.8)	3.6 (1)	3.5 (0.9)	No data	No data	3.6 (1.23)

^*^ Number of respondents varied slightly per question

Our data span from 2008 to 2012. We note a slight increase in scores between 2011 and 2012 in how challenging students perceived the course. This may have been caused by external factors (other things going on in the curriculum at the same time, order of prior course work and training, etc.) or intrinsic student factors. Another difference between 2008 and 2012 regards setting clear expectations for students in this course. In the pilot study (data not shown) [12] students searched RCTs themselves. After the pilot teachers selected the RCTs used in the course, so we improved the quality of used RCTs and the students’ learning experience.

## Discussion

This research-intensive course done by five cohorts of 300 medical students in their second year showed a self-reported increase in learning gain and development of a scientific mindset. Learning gain consists in particular of a better understanding of RCTs and an improved ability to read critically. The scientific quality and authenticity of this research-intensive course done by young students is demonstrated by several publications in journals.

There is evidence that fostering a scientific mindset is possible by doing research at the end of the curriculum [16]. We found no publications describing curricula in which all students conduct real research as early in the curriculum as we describe here. Organizing this research-intensive course was feasible within a very limited time window of 3 weeks by dividing the data collection between all students. This set-up may be applied in other contexts as well, for example involving students in surveys, administering and collecting data in any number of settings (clinical, public schools, health fairs, etc.).

It is a challenge to create the appropriate level of difficulty in a research course for second-year students. In the constructivist paradigm friction between what is known and unknown is an important reason for learning. However, only a constructive friction will facilitate learning, while a destructive friction will work counterproductively by causing fear and uncertainty [17]. This is an important point of attention in implementing such (research) courses. In student-centred education teachers have a guiding role. They shape their role by being clear about learning goals skills in dealing with differences in learning styles and prior knowledge of students in the group.

It would be interesting to further explore the development of scientific skills and mindset of students early in the curriculum. We wondered whether there is a relation between attitude towards research and course grades, for example, also early in the curriculum.

In conclusion it seems possible to implement a research-intensive course using a constructivist approach on learning quite early in the curriculum. Students report it fosters a scientific mindset. Moreover, it has led to publications in peer-reviewed journals, which demonstrates the quality of the work done by the student researchers in this course.

This study has limitations. We acknowledge that our study is based largely on self-reported evaluation data, and no control group was available. However, the response to the questionnaire was high and we have no reason to believe the anonymous responses were biased in any direction.
